# Potentiation of latent inhibition by haloperidol and clozapine is attenuated in Dopamine D2 receptor (Drd-2)-deficient mice: Do antipsychotics influence learning to ignore irrelevant stimuli via *both* Drd-2 and non-Drd-2 mechanisms?

**DOI:** 10.1177/0269881114544774

**Published:** 2014-08-13

**Authors:** Matthew J O’Callaghan, Cecilie Bay-Richter, Colm MP O’Tuathaigh, David M Heery, John L Waddington, Paula M Moran

**Affiliations:** 1 School of Psychology, University of Nottingham, Nottingham, UK; 2 Molecular and Cellular Therapeutics, Royal College of Surgeons in Ireland, Dublin, Ireland; 3 School of Pharmacy, Centre for Biomolecular Sciences, University of Nottingham, Nottingham, UK; 4 Molecular and Cellular Therapeutics, Royal College of Surgeons in Ireland, Dublin, Ireland; + present address: Translational Neuropsychiatry Unit, Department of Clinical Medicine, Aarhus University, Aarhus, Denmark; * present address: School of Medicine, Brookfield Health Sciences Complex, University College Cork, Cork, Ireland

**Keywords:** Antipsychotics, Drd-2 knockout mice, latent inhibition, haloperidol, clozapine

## Abstract

Whether the dopamine Drd-2 receptor is necessary for the behavioural action of antipsychotic drugs is an important question, as Drd-2 antagonism is responsible for their debilitating motor side effects. Using Drd-2 null mice (*Drd_2_* -/-) it has previously been shown that Drd-2 is not necessary for antipsychotic drugs to reverse D-amphetamine disruption of latent inhibition (LI), a behavioural measure of learning to ignore irrelevant stimuli. Weiner’s ‘two-headed’ model indicates that antipsychotics not only reverse LI disruption, ‘disrupted LI’, but also potentiate LI when low/absent in controls, ‘persistent’ LI. We investigated whether antipsychotic drugs haloperidol or clozapine potentiated LI in wild-type controls or *Drd_2_* -/-. Both drugs potentiated LI in wild-type but not in *Drd_2_*-/- mice, suggesting moderation of this effect of antipsychotics in the absence of Drd-2. Haloperidol potentiated LI similarly in both *Drd_1_*-/- and wild-type mice, indicating no such moderation in *Drd_1_*-/-. These data suggest that antipsychotic drugs can have *either* Drd-2 or non-Drd-2 effects on learning to ignore irrelevant stimuli, depending on how the abnormality is produced. Identification of the non-Drd-2 mechanism may help to identify novel non-Drd2 based therapeutic strategies for psychosis.

## Introduction

People with schizophrenia have well-documented attentional impairments, including inability to ignore irrelevant stimuli (Heinrichs and Zakzanis 1998; McGhie and Chapman, 1962; Morris et al., 2013). Consequently, Latent Inhibition (LI), a process of learning to ignore irrelevant stimuli seen in humans and animals, has a long history of application in animal models of the disorder (Lubow, 2010). LI is impaired learning of a conditioned stimulus (CS)–unconditioned stimulus (US) association in a group receiving pre-exposure to that stimulus without reinforcement (pre-exposed, PE), compared with a group without such pre-exposure (non-pre-exposed, NPE). Most studies in patients with schizophrenia, their relatives and psychometrically defined schizotypy find abnormal LI (either inappropriately present or absent depending on symptom profile) (Lubow, 2010, though see Swerdlow, 2010). Based on pharmacological data in rats, Weiner’s ‘two-headed’ model of LI suggests that there are two categories of abnormality in LI that are counteracted by antipsychotic drugs: one in which LI is high in controls and disrupted (e.g. by psychotomimetic D-amphetamine, called ‘disrupted LI’) and one in which it is low in controls and potentiated (e.g. by antipsychotics or psychotomimetics such as scopolamine, MK801 or ketamine, called ‘persistent LI’) (Weiner, 2003; Weiner and Arad, 2009). It has been suggested that disrupted and persistent LI reflect different attentional processes: failure to inhibit attention to irrelevant stimuli and failure to switch attention when previously irrelevant stimuli become relevant, respectively (Weiner and Arad, 2009). Low or no LI is induced experimentally in controls by either increasing the number of CS–US pairings or decreasing the amount of stimulus pre-exposure (Moser et al., 2000; Weiner, 2003; Weiner and Arad, 2009). In humans and rats antipsychotic drugs potentiate low LI induced by decreasing pre-exposure (Moser et al., 2000; Weiner and Arad, 2009). This is also seen in some (though not all) strains of mice, including the C57BL/6 strain used in the present study (Lipina and Roder, 2010; Lipina et al., 2005).

Antipsychotic drugs bind to multiple neurotransmitter and peptide receptors but it is Drd-2 blockade that correlates significantly with clinical potency (Meltzer et al., 1989; Seeman et al., 1976). As all antipsychotic drugs are dopamine receptor Drd-2 antagonists it is inferred that these drugs act via Drd-2; however, it is still unclear whether they are strictly required for all the behavioural effects of antipsychotic drugs. This is an important question, as it is Drd-2 antagonism that produces debilitating extra-pyramidal side effects in patients, which seriously limit compliance (Fleischhacker et al., 1994; Kapur et al., 2000). In models of LI disruption it is inferred that Drd-2 is important in antipsychotic restoration of abnormal LI (Moser et al., 2000; Weiner and Arad, 2009; Weiner, 2003). This is supported by evidence that LI is enhanced in dopamine Drd-2 receptor-deficient mice when LI is low in wild-type controls. This reproduces the behavioural effect of antipsychotic drugs on one of the LI abnormalities described in the ‘two-headed’ model and indirectly suggests a role for Drd-2 in their effects on LI (Bay-Richter et al., 2009). Recently, we found that haloperidol and clozapine attenuate D-amphetamine disruption of LI in mice lacking Drd-2 receptors, suggesting these drugs do not require Drd-2 to reverse D-amphetamine disruption of LI (Bay-Richter et al., 2013). This suggests that the specific behavioural effect of antipsychotic drugs to moderate D-amphetamine disruption of LI can occur without interaction with Drd-2. In the same study using a disrupted LI protocol where LI is present in controls, haloperidol appeared to enhance LI in controls but not mice lacking Drd-2 receptors, though these effects were not statistically supported. This might suggest that, in contrast to reversal of D-amphetamine disruption, antipsychotic drug effects to enhance LI may be moderated in the absence of Drd-2. In the present study we therefore investigated whether antipsychotic drug (haloperidol and clozapine) potentiation of low LI (rendered low in controls by reducing stimulus pre-exposure) is moderated in *Drd_2_*-/- mice. To investigate the specificity to Drd-2 we investigated haloperidol under the same conditions in Dopamine D_1_ receptor (D_1_)-deficient mice (*Drd_1_*-/-).

## Materials and methods

### Mice

Congenic Drd-1 and Drd-2 lines were used at 10–20 weeks of age as described previously (Bay-Richter et al., 2009, 2013). Mice were housed 1–4 per cage on a 12 h light: 12 h dark cycle (lights on 07:00 hr), controlled temperature (20 ± 2°C) and humidity (40–60%), with food ad libitum. Mice were water restricted for 23 h per day throughout the experiment with 1 h free access to water in their home cages after each experimental session. Experiments were carried out in compliance with licence authority under the Animals (Scientific Procedures) Act, UK 1986; UK Home Office Project licence No: 40/2883.

### Genotyping

Genotyping was performed by PCR using genomic DNA extracted from ear biopsies (Bay-Richter et al., 2009).

### Latent inhibition

Latent inhibition was carried out in six identical conditioning chambers (Med-Associate Inc., Vermont, USA) described previously in detail (Bay-Richter et al., 2009, 2013). Briefly, 7 days before testing mice were placed on 23 h water restriction. There then followed 6 days of lick training where mice drank in the chambers for 15 min and the number of licks was recorded. On Day 7 (pre-exposure) mice were placed in the chambers with the waterspout withdrawn. One group received 40 presentations (determined from prior experiments to produce reduced LI in controls) of a 5 s 85 dB tone with a 15 s inter-stimulus interval (pre-exposed group, PE); a control group of non-pre-exposed (NPE) mice were placed in the chambers for an identical period of time but received no tone pre-exposure. On Day 8 (conditioning) mice were placed in chambers with the waterspout withdrawn. After 2 min, two tone–footshock pairings were presented. Tones were of 5 s duration and followed by a 1 s 0.38 mA footshock with an inter-trial interval of 2.5 min. Mice remained in the chamber for 2.5 min following the second shock presentation. On Day 16–17 (rebaseline) mice were placed in chambers for 15 min and given free access to water to re-establish stable licking; the criterion was that mice that did not complete >300 licks continuously did not continue to the test stage (*n*=1). On Day 18 (Test) mice had free access to the waterspout in chambers. Number of licks was recorded and time taken to complete licks 80–90 (A) and 90–100 (B) recorded. After completion of 90 licks, the tone was presented until the mouse reached lick 100 or 600 seconds had elapsed. A suppression ratio (SR) was calculated according to the formula A/ (A + B) yielding a scale of 0–0.5. Low SR indicates increased suppression of drinking; high SR indicates decreased suppression of drinking. LI is seen as higher SR in PE versus NPE groups.

### Experimental design and statistics

Statistics were performed using SPSS (Version 18, 2009 SPSS Inc. Chicago, Illinois, USA). For LI experiments, analysis of variance (ANOVA) was used. For post hoc comparisons, planned comparisons utilized *T*-tests with Bonferroni correction for α slippage. NS indicates not significant. One limitation of the *T*-test approach is that it does not include the error term from the overall ANOVA. We therefore in addition used simple main effects tests using the error term from the main ANOVA following significant three-way interactions and Bonferroni post hoc tests where appropriate. Outliers > 1.5 × inter-quartile range were removed prior to analysis. [*n*=5 exp 1; 2 × Wild-Type NPE Clozapine, 1 × Wild-Type NPE Haloperidol, 1 × *Drd_2_*-/- NPE Haloperidol, 1 × Wild-Type PE vehicle]/[*n*=4 experiment 2; 1 × Wild-Type NPE Vehicle, 1 × Wild-Type NPE Haloperidol, 1 × *Drd_1_*-/- PE haloperidol, 1 × Wild-Type PE haloperidol]. The experiment in *Drd_1_*-/- mice was conducted in females, as we have found male *Drd_1_*-/- mice do not show robust LI (Bay-Richter et al., 2009). Numbers *N* were, experiment 1: 43 (23F, 20M) *Drd–_2_*-/- and 44 (22F, 22M) *Drd–_2_*+/+; experiment 2: 27F *Drd_−1_*+/+ and 26F *Drd_−1_*-/-. In both experiments groups did not differ in time to complete licks 80–90 (time A); in experiment 1 there was no effect of sex or interaction between sex and other variables (all *F* values < 1).

### Drugs and administration

Haloperidol and clozapine (Sigma-Aldrich, Dorset, UK) were dissolved in 25 μL glacial acetic acid and buffered to pH 6.5 using 0.1 mM NaOH prior to final dilution in sterile 0.9% saline to appropriate doses (0.1 mg/kg for haloperidol; 2.5 mg/kg for clozapine), with an injection volume of 10 mL/kg; controls received vehicle to the same injection volumes. Doses of haloperidol and clozapine were maximum doses testable without sedation and based on our previous study (Bay-Richter et al., 2013). Mice received two injections of haloperidol, clozapine or vehicle via intraperitoneal injection, one injection 30 min before pre-exposure and the second 24 h later 30 min before conditioning sessions.

## Results

### Effect of clozapine and haloperidol on low LI in *Drd*_−2_+/+ and *Drd*_−2_-/- mice

**Figure 1.**
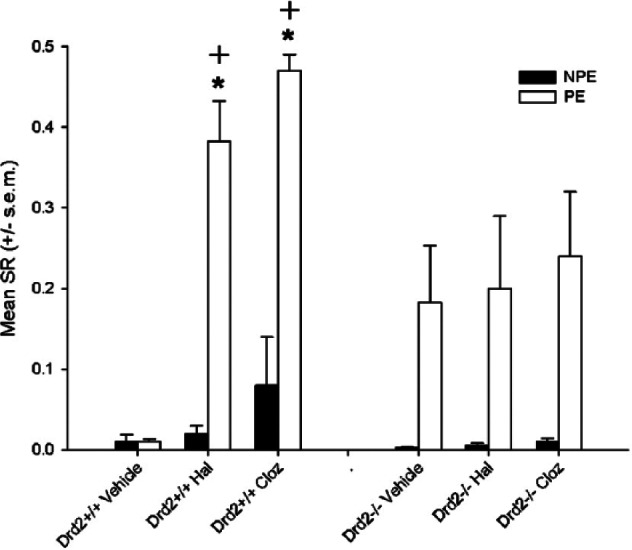
Haloperidol (Hal) and clozapine (Cloz) enhanced LI relative to vehicle controls in *Drd_−2_*+/+ but not *Drd_−2_*-/- mice. Mean suppression ratio (SR) is shown for non-pre-exposed (NPE) and Pre-exposed groups (PE). +indicates *p*<0.05 sig difference from same treatment group in *Drd_−2_*-/- genotype. *indicates *p*<0.05 significant difference from NPE group same treatment group and genotype. A high SR indicates lower suppression a low SR higher suppression.

Both clozapine and haloperidol potentiated LI in *Drd*_−2_+/+ but not in *Drd*_−2_-/- (Figure 1(a)). In a three-way ANOVA (pre-exposure × drug treatment × genotype) there was a significant effect of pre-exposure [*F*_(1,70)_=60.3, *p<*0.001] and drug treatment [*F*_(1,70)_=7.35, *p<*0.001], and pre-exposure × drug treatment [*F*_(2,70)_=6.21, *p<*0.005] and genotype × drug treatment interactions [*F*_(2,70)_=4.49, *p*<0.05]. There was a significant effect of drug treatment in *Drd_−2_*+/+ mice [*F*_(2,34)_=24.8, *p*<0.0001] but not in *Drd_−2_*-/- [*F*_(2,36)_=0.1, NS]. The three-way pre-exposure × genotype × drug treatment interaction was significant [*F*_(2,70)_=4.2, *p*<0.05], therefore drug, genotype and pre-exposure were analysed separately for post hoc comparisons. Vehicle-treated (Veh) *Drd–_2_*+/+ mice receiving a low number of pre-exposures did not show LI [NPE vs. PE T_10_=.24] while clozapine [NPE vs. PE T_11_=18.8, *p*<0.0001]- and haloperidol [NPE vs. PE T_14_=5.9, *p*<0.0001]-treated *Drd_−2_*+/+ mice do. NPE vs. PE was not significant for any drug groups in *Drd_−2_*-/- mice following correction, but Veh and Clozapine groups had uncorrected *p*-values of <0.05. Further support for differences between genotypes is provided by comparison of drug treated PE groups for each genotype; Veh vs. Hal [*Drd_−2_*+/+ T_12_=6.47, *p*<0.001; *Drd_−2_*-/- T_13_=.25, NS]. Veh vs. Clozapine [*Drd_−2_*+/+ T_9_=21.3, *p*<0. 0001; *Drd_−2_*-/- T_15_=.5, NS].

There was a significant effect of drug treatment in PE-*Drd_−2_*+/+ (*F*_(2,17)_=22.4, *p*<0.0005) but not in the corresponding *Drd–_2_*-/- group nor either NPE group. Post hoc Bonferroni tests showed significant differences between PE-Vehicle-*Drd_−2_*+/+ and PE-Haloperidol-*Drd–_2_*+/+ (*p*<0.0001) and PE-Vehicle-*Drd_−2_*+/+ and PE-clozapine-*Drd–_2_*+/+ (*p*<0.0001).

Simple effects tests (Bonferroni adjusted) using the error term from the overall ANOVA further support these conclusions. There was a significant effect of genotype in saline PE (*F*_(1,70)_=4.68, *p*<0.05), haloperidol PE (*F*_(1.70)_=4.98, *p*<0.05)and clozapine PE (*F*_(1,70)_=9.45, *p*<0.005) groups but not in any of the corresponding NPE groups. There was a significant effect of drug in *Drd_−2_*+/+ PE (*F*_(2,71)_=16.38, *p*<0.0001) but not in NPE nor either NPE or PE groups in *Drd*_−2_-/-. There was a significant effect of pre-exposure (i.e. LI) in Veh-*Drd*_−2_-/- (*F*_(1,70)_=5.6, *p*<0.0.001) but not Veh-*Drd*_−2_+/+ (*F*<1) and in all other groups Haloperidol-*Drd*_−2_+/+ (*F*_(1,70)_=23.8, *p*<0.0001), Haloperidol*-Drd*_−2_-/- (*F*_(1,70)_=5.6, *p*<0.05), clozapine-*Drd*−2-/- (*F*_(1,70)_=9.2, *p*<0.005), clozapine-*Drd*_−2_+/+ (*F*_(1,70)_=23.9, *p*<0.0001).

### Effect of haloperidol on low LI (40 PE) in *Drd_1_*-/- mice

**Figure 2.**
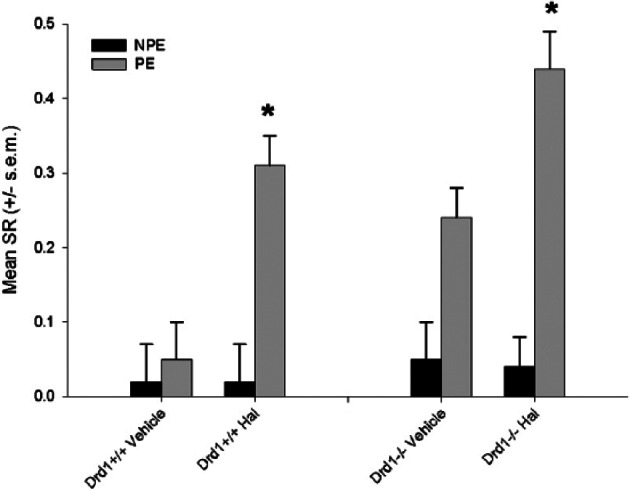
Haloperidol (Hal) enhanced LI in both *Drd–_2_*+/+ and *Drd_−2_*-/- mice. Mean suppression ratio (SR) is shown for non-pre-exposed (NPE) and Pre-exposed groups (PE). *indicates *p*<0.01 significant difference from NPE group same treatment group and genotype. A high SR indicates lower suppression a low SR higher suppression.

Haloperidol significantly potentiated LI (Figure 2). There was a significant effect of genotype [*F*_(1,41)_=6.6, *p*<0.05], Drug treatment [*F*_(1,41)_=9.4, *p*<0.05], pre-exposure [*F*_(1,41)_=38.2, *p*<0.0001] and a significant drug treatment × pre-exposure interaction [*F*_(1,41)_=6.1, *p*<0.01]. This interaction allowed us to examine the effect of drug on NPE and PE separately. There was a significant effect of drug treatment in the PE [*F*_(1,23)_=8.0, *p*<0.001] and not the NPE [*F*=.1, NS] group. There was a significant effect of pre-exposure (i.e. LI) in Haloperidol (F_(1,24)_=30.4, *p*<0.0001) but not vehicle-treated groups. There was no indication that haloperidol induction of LI was moderated in *Drd−1*-/- mice (drug treatment × genotype interaction [F=.22, NS]). NPE vs. PE comparisons were significant for Hal-treated groups [*Drd_−1_*+/+ T_(11)_=3.2, *p*<0.01; *Drd_−1_*-/- T_(10)_=15.4, *p*<0.001] but not vehicle-treated groups [*Drd_−1_*+/+ T_(9)_=0.9; *Drd_−1_*-/- T_(11)_=2.0, NS].

## Discussion

Clozapine and haloperidol potentiated LI relative to vehicle-treated controls in *Drd_−2_*+/+ mice; *Drd_−2_*-/- mice showed similar LI whether they received vehicle, haloperidol or clozapine. The effects of haloperidol and clozapine on pre-exposure were significantly lower in *Drd_−2_*-/- compared with *Drd_−2_*+/+ controls (Figure 1). This suggests that lack of Drd-2 receptors may moderate the effect of these drugs to potentiate LI. Taken in conjunction with recent findings that Drd-2 is not necessary for antipsychotics to reverse D-amphetamine disruption of LI (Bay-Richter et al., 2013), the present data suggest that antipsychotics can restore abnormal LI via *either* a Drd-2 or a non-Drd-2 mechanism depending on how the abnormality is induced.

*Drd_−2_*-/- mice showed enhanced LI relative to controls; this is consistent with previous experiments in these mice, and mimics effects of antipsychotic drugs given alone (Bay-Richter et al., 2009; Moser et al., 2000). One possible explanation for the failure to observe potentiated LI by antipsychotics in *Drd_−2_*-/- is that they already show the maximum LI attainable, and in these conditions further enhancement would normally no longer be detectable (Bay-Richter et al., 2009). This is an unlikely explanation as we observe drug effects in the same experiment in *Drd–_2_*+/+ to have produced much higher PE values, suggesting further enhancement is detectable. Furthermore, there is clear dissociation from the effect of haloperidol in *Drd_−1_*-/- in experiment 2, which is comparable between *Drd_−1_*+/+ and *Drd_−1_*-/- mice.

These data are consistent with theories of schizophrenia that suggest antipsychotic drugs act via Drd-2 to modify processes that allocate salience based on prior experience (Gray et al., 1991; Kapur 2003, 2004; Kapur et al., 2005). One possible conceptualisation of antipsychotic drug effects to potentiate LI where it is absent in controls, is that they reduce the salience of whatever is demanding most attention currently. Where a tone is associated with either no consequence or footshock, footshock will normally demand attention when the number of pre-exposures is low. In this case, antipsychotic drugs reduce the relative salience of the tone–footshock relative to the tone–no consequence association. This is not simply a disruption of the tone–footshock pairing as NPE groups are unaffected. It has been suggested that in patients antipsychotics reduce the salience of positive symptoms (Kapur et al., 2005), thus if a particular percept or hallucination were currently demanding the most attention then it would be predicted that antipsychotics would reduce its salience. Theoretical models such as the switching hypothesis have proposed explanations of how antipsychotic drugs affect these LI phenomena (Weiner and Arad, 2009). These data suggest that Drd-2 antagonism may be important for this salience-modulating action of the antipsychotic drugs haloperidol and clozapine at doses tested, though generalisation to other behavioural models of these processes is merited to assess this more specifically. The suggestion that Drd-2 is important is consistent with experimental predictions from pharmacological studies of LI in rats, mice and humans (Weiner and Arad, 2009; Kumari et al., 1999; Moser et al., 2000) but contrasts with effects of these drugs in D-amphetamine-disrupted LI which are not Drd-2 dependent (Bay-Richter et al., 2013). That finding was difficult to reconcile with the well-established correlation between affinity for the Drd-2 receptor and clinical efficacy of antipsychotics. The present study now shows that lack of Drd-2 can moderate the effect of antipsychotics depending on which ‘head’ of the LI model is being investigated, ‘disrupted’ or ‘persistent’. This suggests that these antipsychotic drugs may have two behavioural actions, one possibly requiring Drd-2 and one not. One behavioural effect is unmasked under conditions of high DA (or other neurotransmitter) release following D-amphetamine where LI is high in controls and is not Drd-2 dependent (Bay-Richter et al., 2013), another where LI is low in controls and is moderated in the absence of Drd-2.

*Drd_1_*-/- mice show a trend towards potentiated LI relative to controls, again consistent with our previous findings in female mice (Bay-Richter et al., 2009). Haloperidol enhanced LI relative to controls in both *Drd_−1_*+/+ and *Drd_−1_*-/- mice. This suggests that lack of *Drd_−1_* does not moderate the effect of haloperidol to potentiate low LI. Pharmacological and null mouse studies have previously suggested that Drd-1 might play a role in D-amphetamine disruption of LI and consequently in the effects of antipsychotics to reverse these effects (Bay-Richter et al., 2013; Nelson et al, 2012). Our findings suggest that this may not apply to the potentiation of low LI. Our data are, however, consistent with previous findings that antipsychotic drugs that are antagonists at both Drd-1 and Drd-2, reverse D-amphetamine-disrupted LI and enhance LI when it is low in controls. Conversely, selective Drd-1 antagonists reverse amphetamine-disrupted LI but do not enhance low LI (Trimble et al., 2002). This potentially reconciles strong clinical evidence that Drd-2 is important for the effects of antipsychotic drugs with arguments that Drd-1 antagonism may play a role in the behavioural effects of antipsychotics (e.g. Miller, 2009a, 2009b). By necessity the Drd-1 experiment was carried out in females; there remains a possibility that in males the Drd-1 dependence of haloperidol may differ. Because of baseline differences this is not testable using this null mouse approach, though experiments in male rats suggest that low LI is not enhanced by selective Drd-1 antagonists (Trimble et al, 2002; Nelson et al., 2012).

We have demonstrated that antipsychotic drug potentiation of LI is reduced in mice lacking Drd-2. Taken together with previous findings that antipsychotics can attenuate D-amphetamine disruption of LI in mice lacking Drd-2, the data suggest that antipsychotic drugs may restore abnormality in learning to ignore irrelevant stimuli via *either* Drd-2 dependent or independent mechanisms. Identification of the non-Drd-2 mechanism through which antipsychotics specifically modulate salience but not other behaviours may help to identify novel non-Drd-2 based therapeutic strategies for psychosis.
